# Supplementary figures and images for: Spatial risk modelling of highly pathogenic avian influenza in France: Fattening duck farm activity matters
Source: PLoS One. 2025 Feb 4;20(2):e0316248. doi: 10.1371/journal.pone.0316248 (PMC11793745; doi:10.1371/journal.pone.0316248)

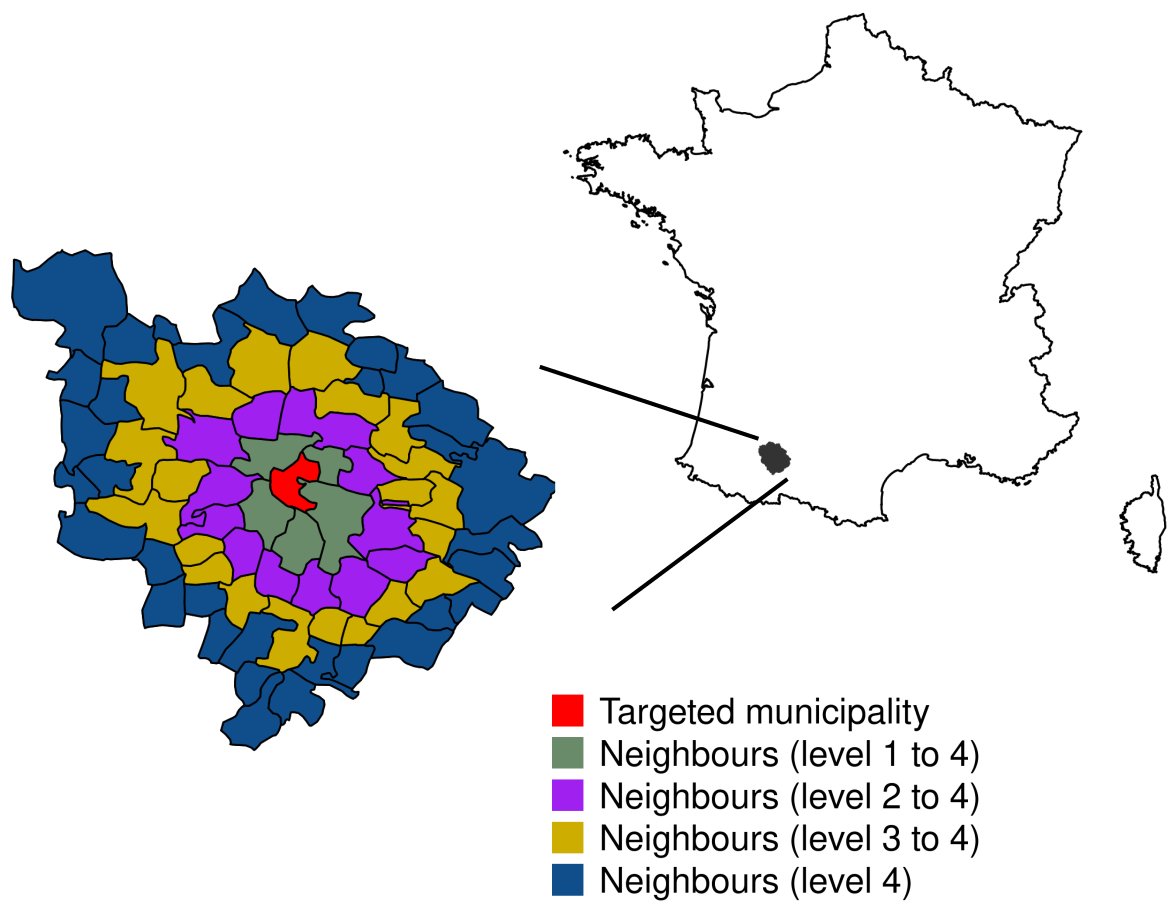

Supplement: S1 Fig — Spatial representation of a given municipality and neighboring municipalities across four spatial scales, from the level 1 (olive green) to the level 4 (dark blue). Shapefiles used to create maps are based on administrative boundaries available in the public domain (CC BY 4.0). (TIF) [file pone.0316248.s001.tif]

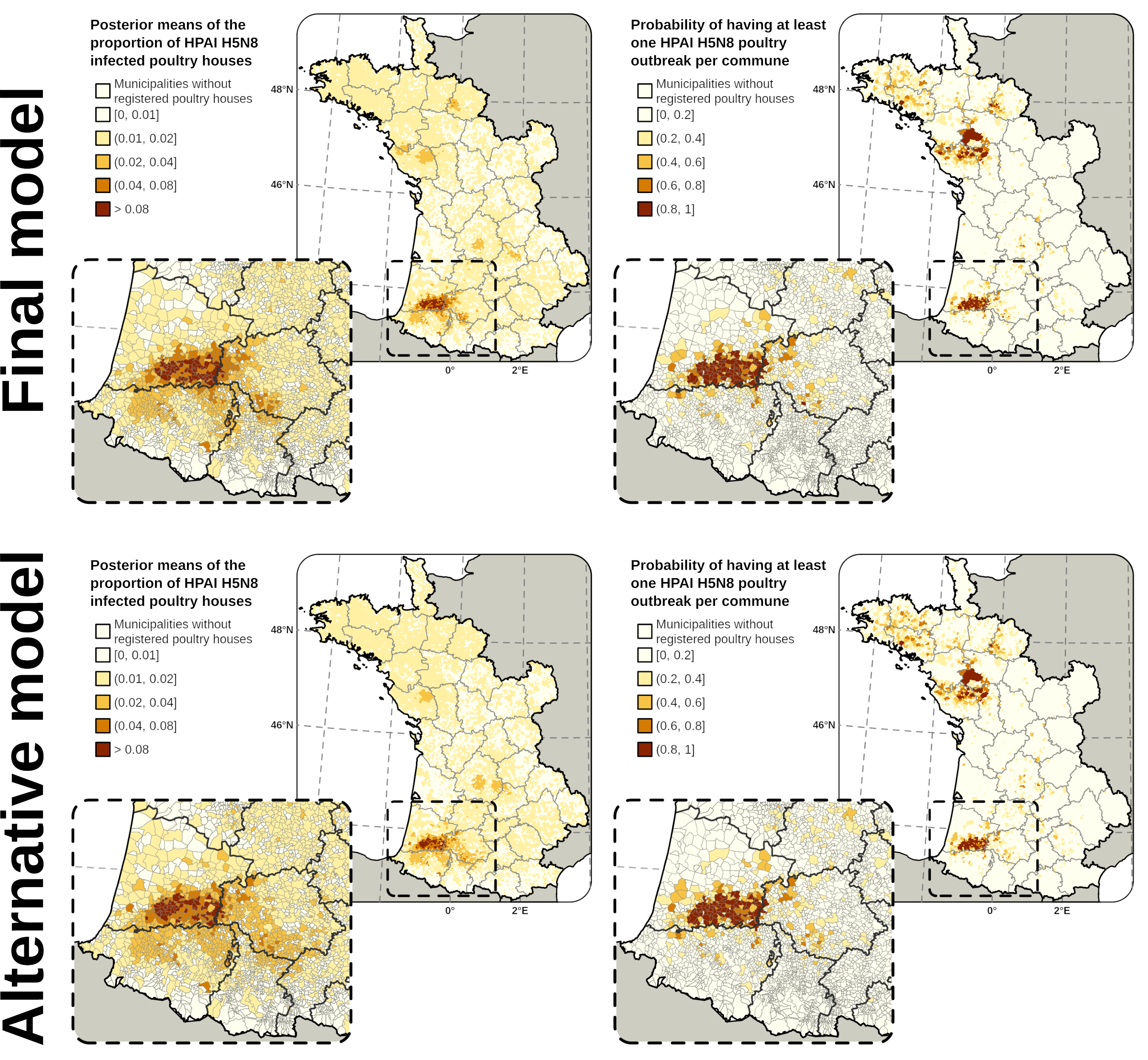

Supplement: S2 Fig — The final model results are represented on top of the figure and the alternative model results are in the bottom part of the figure. The posterior means of the proportion of HPAI H5N8 infected poultry houses per municipality are represented on the left panels and the probability of having at least one HPAI H5N8 poultry outbreak per commune are represented on the right panels. Shapefiles used to create maps are based on administrative boundaries available in the public domain (CC BY 4.0). (TIF) [file pone.0316248.s002.tif]
