# Supplementary material for: Spatial risk modelling of highly pathogenic avian influenza in France: Fattening duck farm activity matters
Source: PLoS One. 2025 Feb 4;20(2):e0316248. doi: 10.1371/journal.pone.0316248 (PMC11793745; doi:10.1371/journal.pone.0316248)
Supplement: S1 File — Predictor variables included the number of poultry (chicken and duck) houses, the density of chicken houses per km2, the density of duck houses per km2, the density of fattening duck (all stages) houses per km2, the density of fattening duck (breeding stage) houses per km2 and the density of fattening duck (force-feeding stage) houses per km2. Shapefiles used to create maps are based on administrative boundaries available in the public domain (CC BY 4.0). (PDF) [file pone.0316248.s003.pdf]

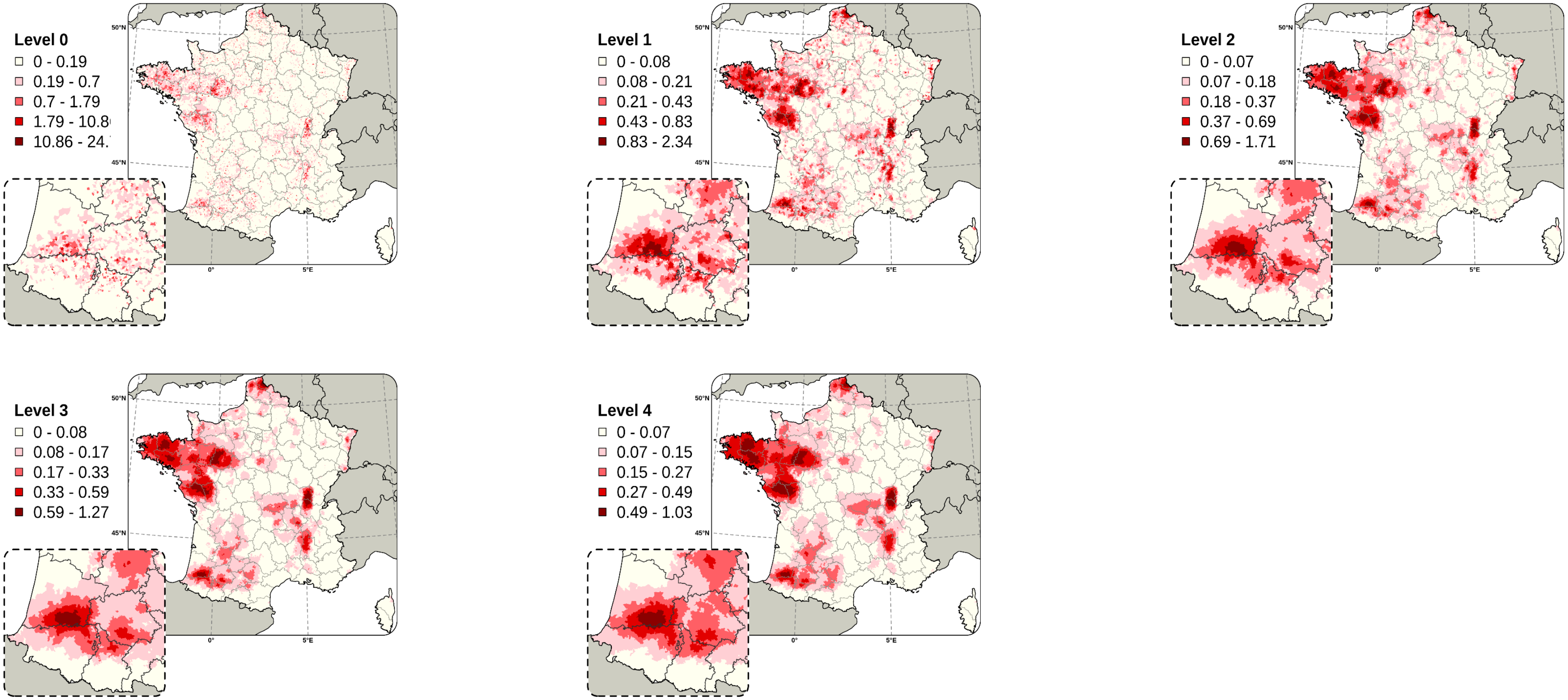


**S1 File. Spatial distribution of predictor variables.** Density of chicken houses (/km2).

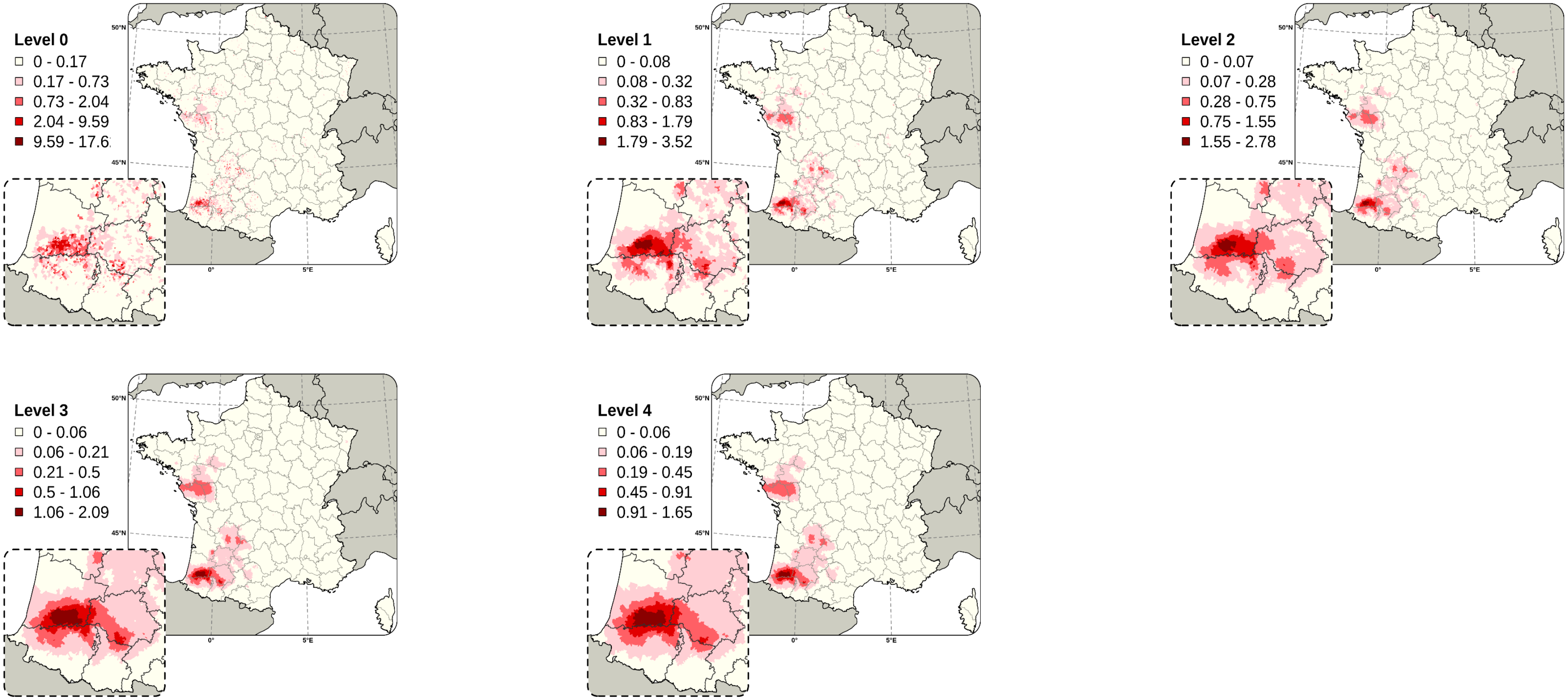


**S1 File. Spatial distribution of predictor variables.** Density of duck houses (/km2)

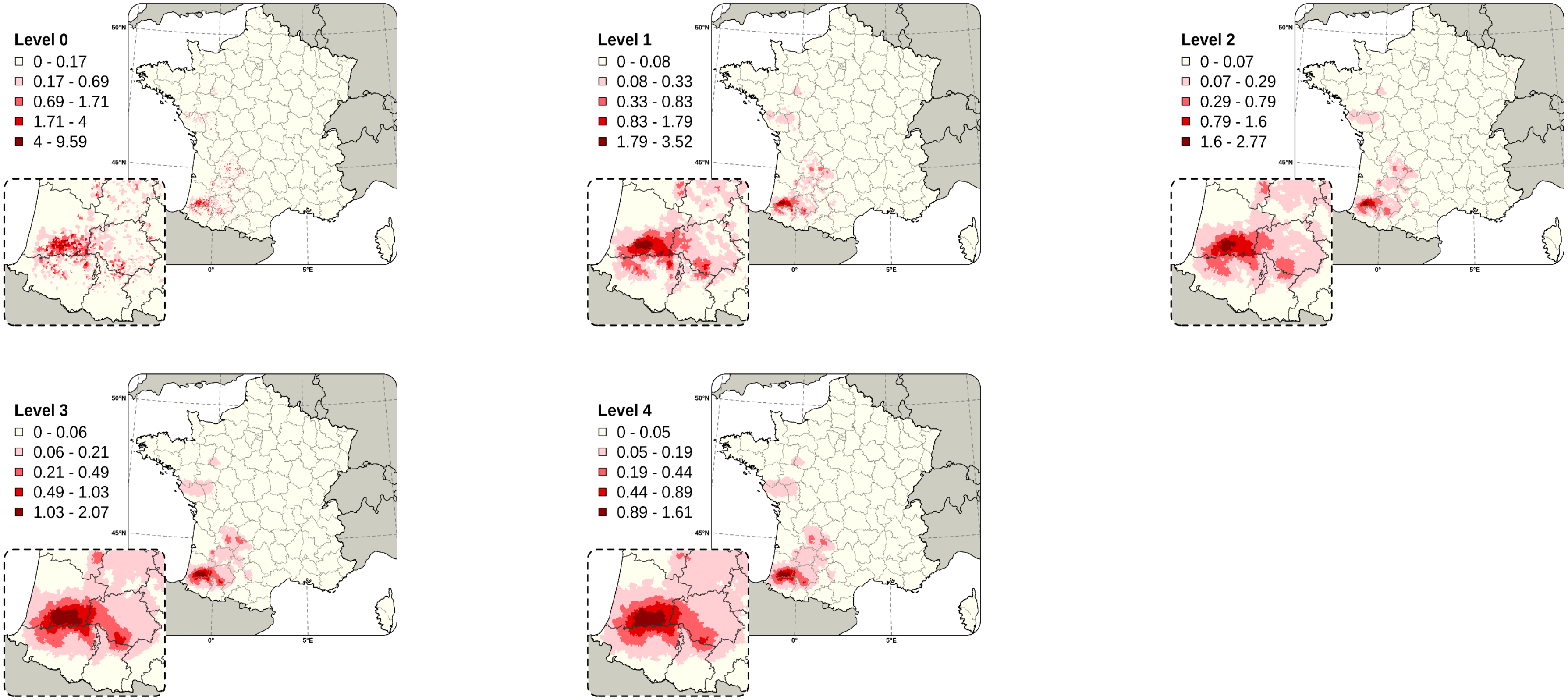


**S1 File. Spatial distribution of predictor variables.** Density of fattening duck (all stages) houses (/km2).

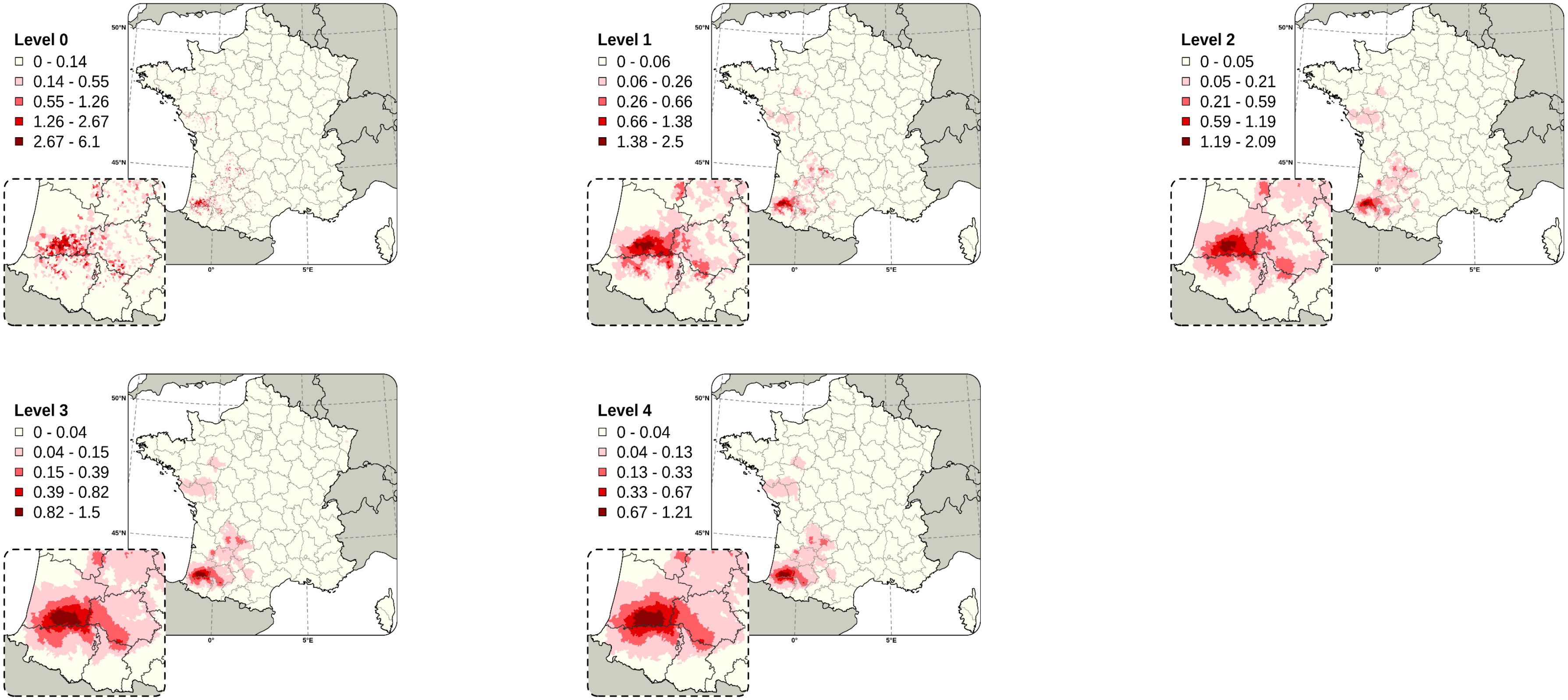


**S1 File. Spatial distribution of predictor variables.** Density of fattening duck (breeding stage) houses (/km2).

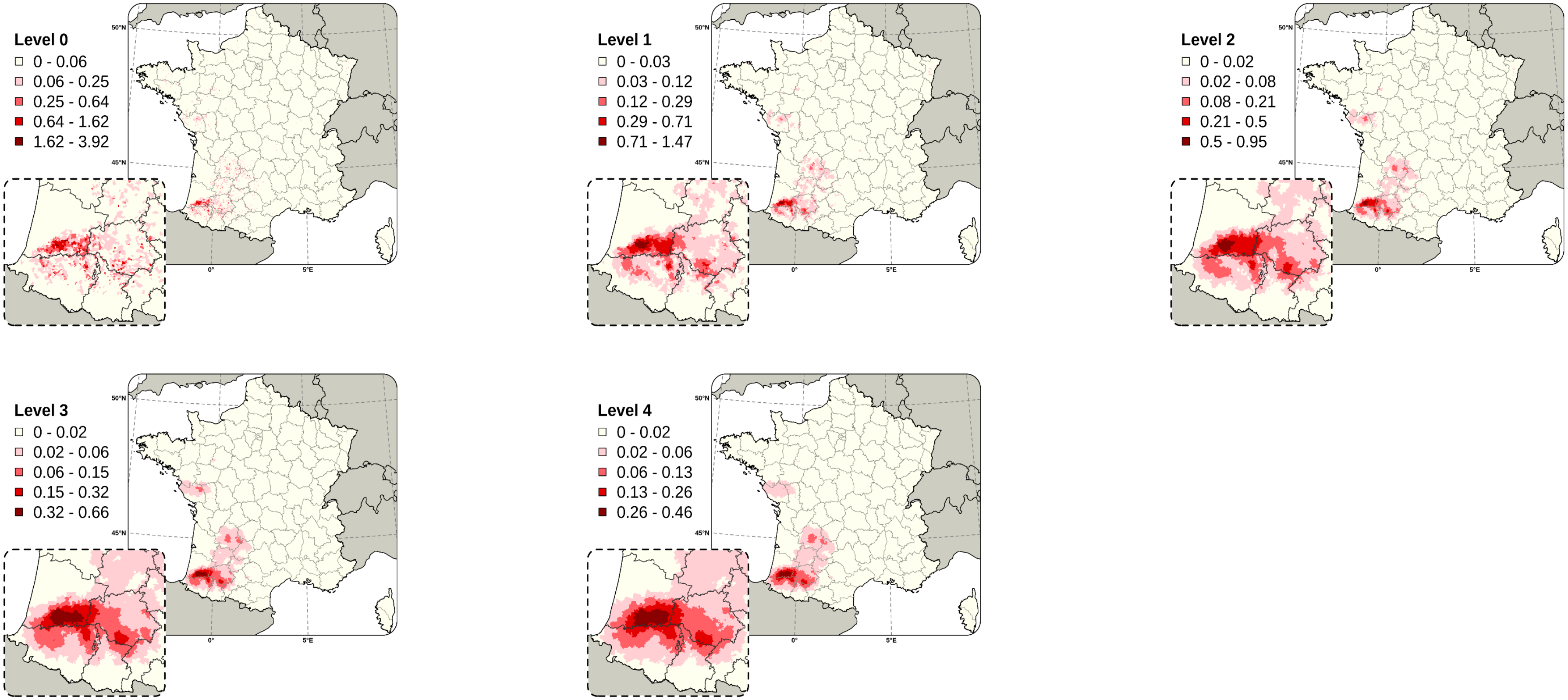


**S1 File. Spatial distribution of predictor variables.** Density of fattening duck (force-feeding stage) houses (/km2).

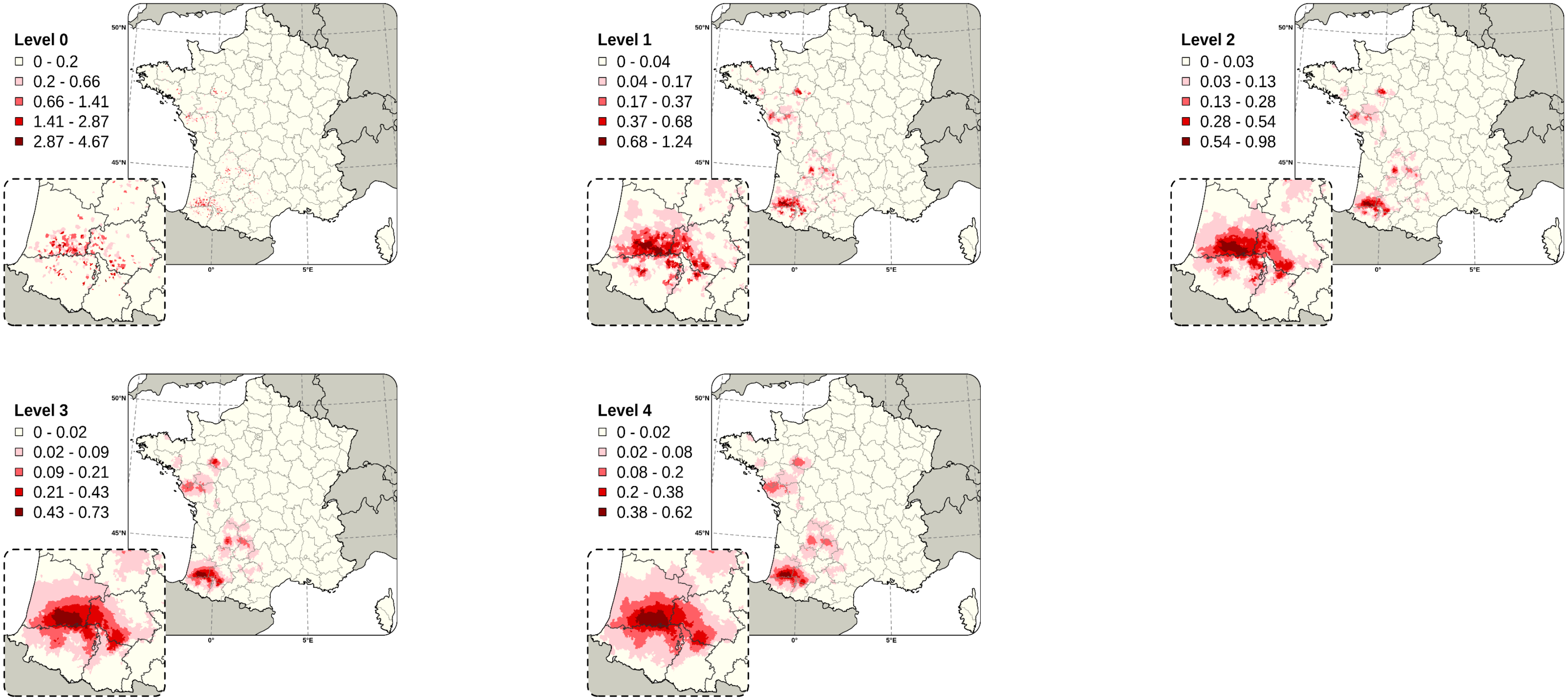


**S1 File. Spatial distribution of predictor variables.** Density of outgoing fattening duck flock movements (breeding to force-feeding stage) (/km2).

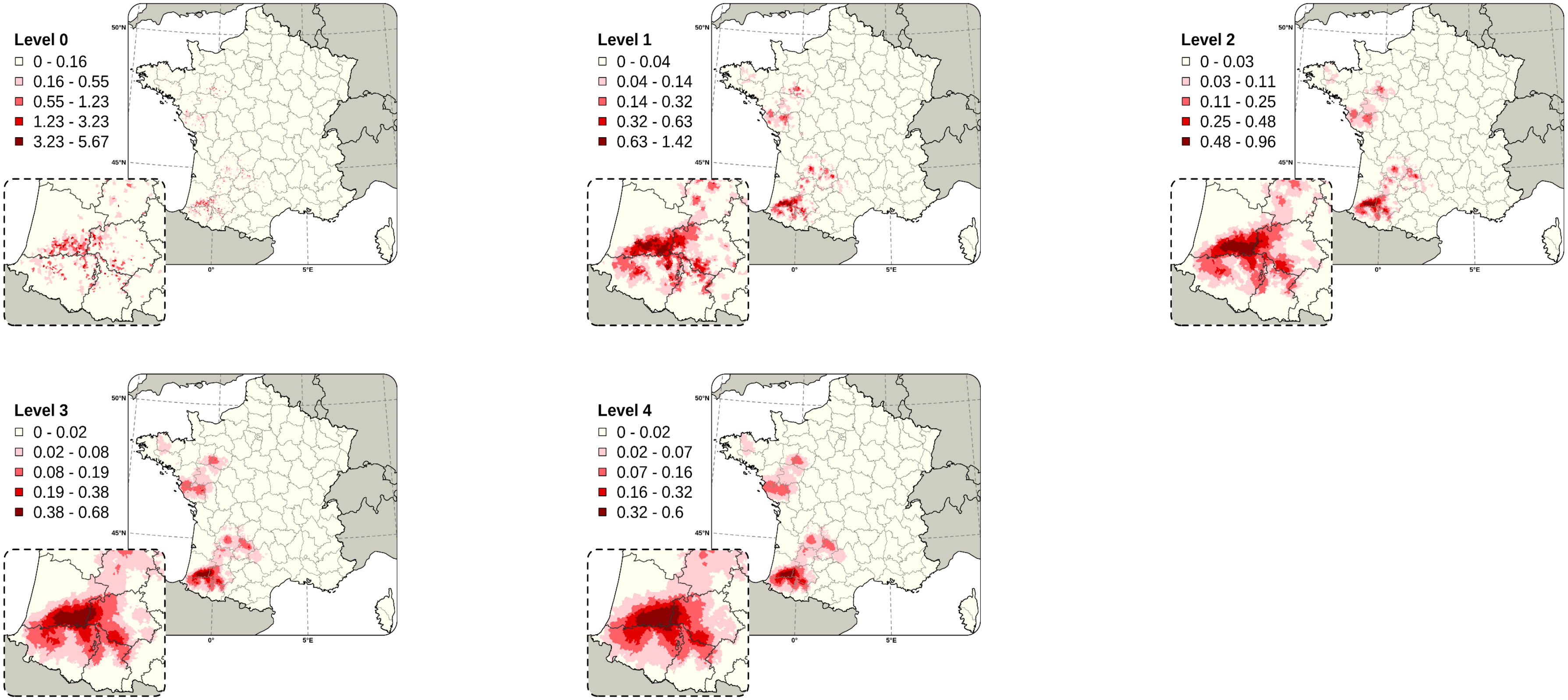


**S1 File. Spatial distribution of predictor variables.** Density of incoming fattening duck flock movements (breeding to force-feeding stage) (/km2).

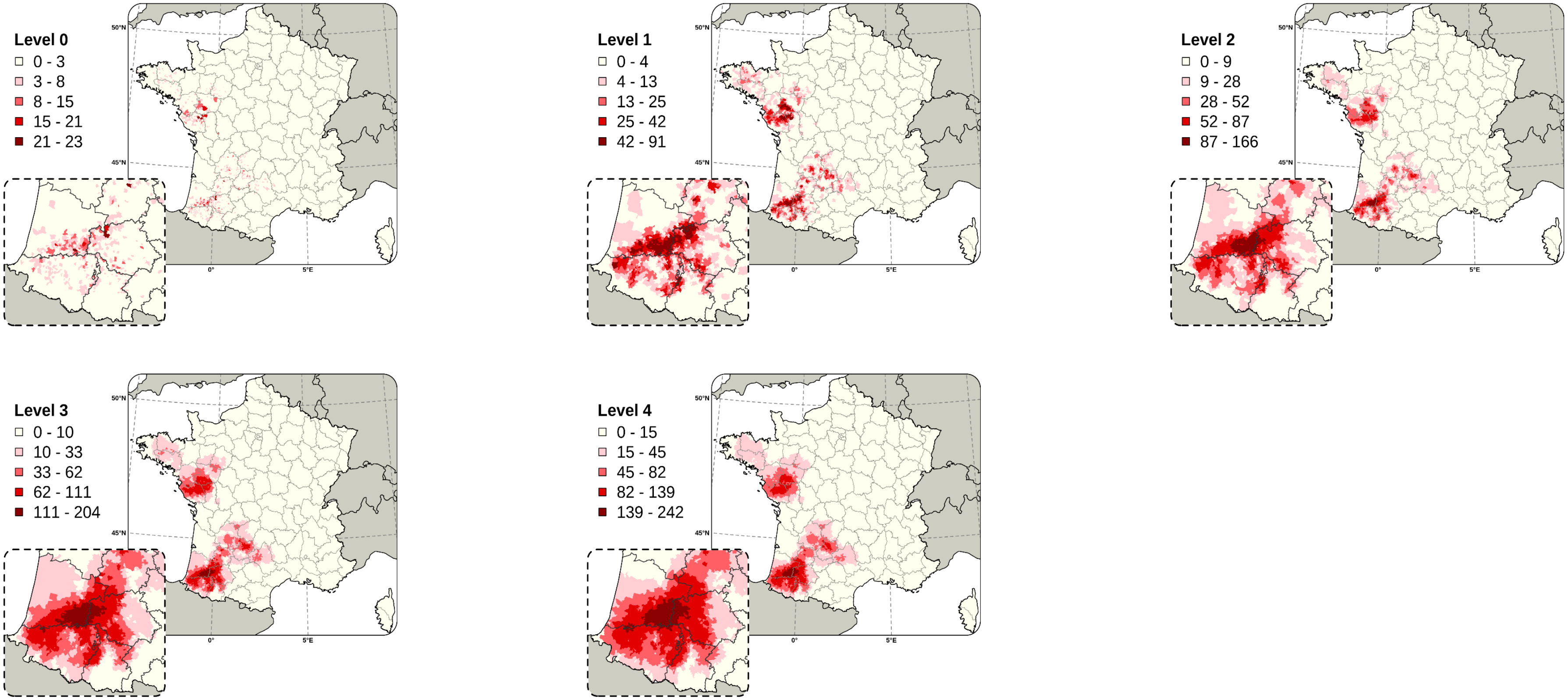


**S1 File. Spatial distribution of predictor variables.** Number of municipalities sending fattening duck flocks to the given municipality (breeding to force-feeding stage).
