# Supplementary material for: Spatial risk modelling of highly pathogenic avian influenza in France: Fattening duck farm activity matters
Source: PLoS One. 2025 Feb 4;20(2):e0316248. doi: 10.1371/journal.pone.0316248 (PMC11793745; doi:10.1371/journal.pone.0316248)
Supplement: Attachment — Submitted filename: Response to Reviewers.docx [file pone.0316248.s007.docx]

Journal Requirements:

When submitting your revision, we need you to address these additional requirements.

1. Please ensure that your manuscript meets PLOS ONE's style requirements, including those for file naming. The PLOS ONE style templates can be found at 
<https://journals.plos.org/plosone/s/file?id=wjVg/PLOSOne_formatting_sample_main_body.pdf> and 
<https://journals.plos.org/plosone/s/file?id=ba62/PLOSOne_formatting_sample_title_authors_affiliations.pdf>

The manuscript has been updated according to the journal requirements

2. Please include a complete copy of PLOS’ questionnaire on inclusivity in global research in your revised manuscript. Our policy for research in this area aims to improve transparency in the reporting of research performed outside of researchers’ own country or community. The policy applies to researchers who have travelled to a different country to conduct research, research with Indigenous populations or their lands, and research on cultural artefacts. The questionnaire can also be requested at the journal’s discretion for any other submissions, even if these conditions are not met.  Please find more information on the policy and a link to download a blank copy of the questionnaire here: <https://journals.plos.org/plosone/s/best-practices-in-research-reporting>. Please upload a completed version of your questionnaire as Supporting Information when you resubmit your manuscript.

The following sentence has been added to the Methods: “Additional information regarding the ethical, cultural, and scientific considerations specific to inclusivity in global research is included in the Supporting Information (S1 Checklist)”

The questionnaire has been added to the Supporting Information.

3. Please note that PLOS ONE has specific guidelines on code sharing for submissions in which author-generated code underpins the findings in the manuscript. In these cases, we expect all author-generated code to be made available without restrictions upon publication of the work. Please review our guidelines at [https://journals.plos.org/plosone/s/materials-and-software-sharing#loc-sharing-code](_blank) and ensure that your code is shared in a way that follows best practice and facilitates reproducibility and reuse.

The code has been made available on https://zenodo.org/records/13765228 (DOI 10.5281/zenodo.13765227). The following sentence has been added tot the Methods: “The R code about the spatial smoothing of variables is available online (10.5281/zenodo.13765228).”

4. Please note that funding information should not appear in any section or other areas of your manuscript. We will only publish funding information present in the Funding Statement section of the online submission form. Please remove any funding-related text from the manuscript.

The funding-related text has been removed from the manuscript.

5. Thank you for stating the following financial disclosure: 
 "This study was performed in the framework of the Chair for Avian Biosecurity and Health, hosted by the National Veterinary College of Toulouse and funded by the Direction Générale de l’Alimentation, Ministère de l’Agriculture et de l’Alimentation, France. Jean Artois and Simon Dellicour have received funding from the European Union’s Horizon 2020 research and innovation programme under grant agreement No 874850. The contents of this publication are the sole responsibility of the authors and don't necessarily reflect the views of the European Commission. Simon Dellicour also acknowledges support from the Fonds National de la Recherche Scientifique (F.R.S.-FNRS, Belgium; grant n°F.4515.22) and from the Research Foundation - Flanders (Fonds voor Wetenschappelijk Onderzoek-Vlaanderen, FWO, Belgium; grant n°G098321N)."

Please state what role the funders took in the study.  If the funders had no role, please state: "The funders had no role in study design, data collection and analysis, decision to publish, or preparation of the manuscript." 
If this statement is not correct you must amend it as needed. 
Please include this amended Role of Funder statement in your cover letter; we will change the online submission form on your behalf.

The following sentence has been added to the funding statement: “The funders had no role in study design, data collection and analysis, decision to publish, or preparation of the manuscript.”

6. We note that you have indicated that there are restrictions to data sharing for this study. PLOS only allows data to be available upon request if there are legal or ethical restrictions on sharing data publicly. For more information on unacceptable data access restrictions, please see [http://journals.plos.org/plosone/s/data-availability#loc-unacceptable-data-access-restrictions](_blank).

Before we proceed with your manuscript, please address the following prompts:

a) If there are ethical or legal restrictions on sharing a de-identified data set, please explain them in detail (e.g., data contain potentially identifying or sensitive patient information, data are owned by a third-party organization, etc.) and who has imposed them (e.g., a Research Ethics Committee or Institutional Review Board, etc.). Please also provide contact information for a data access committee, ethics committee, or other institutional body to which data requests may be sent.
b) If there are no restrictions, please upload the minimal anonymized data set necessary to replicate your study findings to a stable, public repository and provide us with the relevant URLs, DOIs, or accession numbers. For a list of recommended repositories, please see
   <https://journals.plos.org/plosone/s/recommended-repositories>. You also have the option of uploading the data as Supporting Information files, but we would recommend depositing data directly to a data repository if possible.

We will update your Data Availability statement on your behalf to reflect the information you provide.

Information about data sharing has been clarified in the Data Availability Statement: “Detailed data about the poultry farm densities and poultry trade used in this study are not publicly accessible due to sensitive farmer information and are owned by the Direction Générale de l’Alimentation, Ministère de l’Agriculture et de l’Alimentation (DGAl) of the Ministère de l’Agriculture et de la Souveraineté Alimentaire, France. Data requests can be made to the DGAl. Data about poultry outbreaks can be found on the Global Animal Disease Information System (https://empres-i.apps.fao.org) of the Food and Agriculture Organization of the United Nations (FAO). Shapefiles used to create maps can be obtained from <https://www.data.gouv.fr/fr/datasets/admin-express/> under the Etalab Open License (equivalent to CC-BY 2.0) from the Institut National de l’Informaiton Géographique et Forestiere (IGN). The Global Administrative Unit Layers (GAUL) dataset was also used (Urbano, Ferdinando (2018): Global administrative boundaries. European Commission, Joint Research Centre (JRC) [Dataset] PID: http://data.europa.eu/89h/jrc-10112-10004). The code has been made available on https://zenodo.org/records/13765228 (DOI 10.5281/zenodo.13765227).

7. We note that Figures 1-3 and Supplementary figures 1-3 in your submission contain map/satellite images which may be copyrighted. All PLOS content is published under the Creative Commons Attribution License (CC BY 4.0), which means that the manuscript, images, and Supporting Information files will be freely available online, and any third party is permitted to access, download, copy, distribute, and use these materials in any way, even commercially, with proper attribution. For these reasons, we cannot publish previously copyrighted maps or satellite images created using proprietary data, such as Google software (Google Maps, Street View, and Earth). For more information, see our copyright guidelines: <http://journals.plos.org/plosone/s/licenses-and-copyright>.
 
We require you to either (a) present written permission from the copyright holder to publish these figures specifically under the CC BY 4.0 license, or (b) remove the figures from your submission:
 
a. You may seek permission from the original copyright holder of Figures 1-3 and Supplementary figures 1-3 to publish the content specifically under the CC BY 4.0 license.  
 
We recommend that you contact the original copyright holder with the Content Permission Form (<http://journals.plos.org/plosone/s/file?id=7c09/content-permission-form.pdf>) and the following text:
“I request permission for the open-access journal PLOS ONE to publish XXX under the Creative Commons Attribution License (CCAL) CC BY 4.0 (<http://creativecommons.org/licenses/by/4.0/>). Please be aware that this license allows unrestricted use and distribution, even commercially, by third parties. Please reply and provide explicit written permission to publish XXX under a CC BY license and complete the attached form.”
 
Please upload the completed Content Permission Form or other proof of granted permissions as an "Other" file with your submission.
 
In the figure caption of the copyrighted figure, please include the following text: “Reprinted from [ref] under a CC BY license, with permission from [name of publisher], original copyright [original copyright year].”

b. If you are unable to obtain permission from the original copyright holder to publish these figures under the CC BY 4.0 license or if the copyright holder’s requirements are incompatible with the CC BY 4.0 license, please either i) remove the figure or ii) supply a replacement figure that complies with the CC BY 4.0 license. Please check copyright information on all replacement figures and update the figure caption with source information. If applicable, please specify in the figure caption text when a figure is similar but not identical to the original image and is therefore for illustrative purposes only.
The following resources for replacing copyrighted map figures may be helpful:
 
USGS National Map Viewer (public domain): <http://viewer.nationalmap.gov/viewer/>
The Gateway to Astronaut Photography of Earth (public domain): <http://eol.jsc.nasa.gov/sseop/clickmap/>
Maps at the CIA (public domain): <https://www.cia.gov/library/publications/the-world-factbook/index.html> and <https://www.cia.gov/library/publications/cia-maps-publications/index.html>
NASA Earth Observatory (public domain): <http://earthobservatory.nasa.gov/>
Landsat: <http://landsat.visibleearth.nasa.gov/>
USGS EROS (Earth Resources Observatory and Science (EROS) Center) (public domain): [http://eros.usgs.gov/#](http://eros.usgs.gov/)
Natural Earth (public domain): <http://www.naturalearthdata.com/>

We are not using copyrighted map figures. Shapefiles used to create maps were obtained from <https://www.data.gouv.fr/fr/datasets/admin-express/> under the Etalab Open Licence (https://www.etalab.gouv.fr/wp-content/uploads/2014/05/Licence_Ouverte.pdf) (equivalent to CC-BY 2.0) from the Institut National de l’Informaiton Géographique et Forestiere (IGN). We are also using the Global Administrative Unit Layers (GAUL) dataset (Urbano, Ferdinando (2018): Global administrative boundaries. European Commission, Joint Research Centre (JRC) [Dataset] PID: http://data.europa.eu/89h/jrc-10112-10004). The following sentence has been added to each figure caption: “*Shapefiles used to create maps are based on administrative boundaries available in the public domain.*”

8. Please include captions for your Supporting Information files at the end of your manuscript, and update any in-text citations to match accordingly. Please see our Supporting Information guidelines for more information: <http://journals.plos.org/plosone/s/supporting-information>.

The captions have been added at the end of the manuscript:
“Supporting Information

**S1 Fig. Smoothing of the spatial predictors.** Spatial representation of a given municipality and neighboring municipalities across four spatial scales, from the level 1 (olive green) to the level 4 (dark blue). *Shapefiles used to create maps are based on administrative boundaries available in the public domain.*

**S1 File. Spatial distribution of predictor variables.** Predictor variables included the number of poultry (chicken and duck) houses, the density of chicken houses per km^2^, the density of duck houses per km^2^, the density of fattening duck (all stages) houses per km^2^, the density of fattening duck (breeding stage) houses per km^2^ and the density of fattening duck (force-feeding stage) houses per km^2^. *Shapefiles used to create maps are based on administrative boundaries available in the public domain.*

**S2 Fig. Comparison of high-risk areas obtained using different statistical models.** The final model results are represented on top of the figure and the alternative model results are in the bottom part of the figure. The posterior means of the proportion of HPAI H5N8 infected poultry houses per municipality are represented on the left panels and the probability of having at least one HPAI H5N8 poultry outbreak per commune are represented on the right panels. *Shapefiles used to create maps are based on administrative boundaries available in the public domain.*

**S1 Table. Summary of the final binomial iid models fits.**

9. Please review your reference list to ensure that it is complete and correct. If you have cited papers that have been retracted, please include the rationale for doing so in the manuscript text, or remove these references and replace them with relevant current references. Any changes to the reference list should be mentioned in the rebuttal letter that accompanies your revised manuscript. If you need to cite a retracted article, indicate the article’s retracted status in the References list and also include a citation and full reference for the retraction notice.

The reference list has been updated.

[Note: HTML markup is below. Please do not edit.]

Reviewers' comments:

Reviewer's Responses to Questions

**Comments to the Author**

1. Is the manuscript technically sound, and do the data support the conclusions?

The manuscript must describe a technically sound piece of scientific research with data that supports the conclusions. Experiments must have been conducted rigorously, with appropriate controls, replication, and sample sizes. The conclusions must be drawn appropriately based on the data presented.

Reviewer #1: Yes

Reviewer #2: Yes

2. Has the statistical analysis been performed appropriately and rigorously?

Reviewer #1: Yes

Reviewer #2: Yes

3. Have the authors made all data underlying the findings in their manuscript fully available?

The [PLOS Data policy](http://c05y1x9s.r.us-east-2.awstrack.me/L0/http:%2F%2Fwww.plosone.org%2Fstatic%2Fpolicies.action%23sharing/1/010f01919ab630f5-7518b5e4-070a-4566-a57b-e47ac9bbefc6-000000/IuqUZXqjUYnzY_1HFdHmjzKx8_U=174) requires authors to make all data underlying the findings described in their manuscript fully available without restriction, with rare exception (please refer to the Data Availability Statement in the manuscript PDF file). The data should be provided as part of the manuscript or its supporting information, or deposited to a public repository. For example, in addition to summary statistics, the data points behind means, medians and variance measures should be available. If there are restrictions on publicly sharing data—e.g. participant privacy or use of data from a third party—those must be specified.

Reviewer #1: Yes

Reviewer #2: Yes

4. Is the manuscript presented in an intelligible fashion and written in standard English?

PLOS ONE does not copyedit accepted manuscripts, so the language in submitted articles must be clear, correct, and unambiguous. Any typographical or grammatical errors should be corrected at revision, so please note any specific errors here.

Reviewer #1: Yes

Reviewer #2: Yes

5. Review Comments to the Author

Please use the space provided to explain your answers to the questions above. You may also include additional comments for the author, including concerns about dual publication, research ethics, or publication ethics. (Please upload your review as an attachment if it exceeds 20,000 characters)

Reviewer #1: In summary, the research provides valuable insights into the spatial risk factors and predictive models for HPAI infection in France.
The study's focus on accessible data and high-risk areas makes it highly relevant for practical applications in disease control.
Recommendations for future research could include exploring additional predictor variables or applying the model to other regions or types of poultry.
This analysis suggests that the research is well-positioned to contribute to both scientific understanding and practical efforts to manage HPAI, with some room for improvement in the areas of methodological transparency and supplementary materials.

Recommendations about future research has been clarified: “In our explanatory work, we also considered various potential variables, including farm biosecurity levels or staff and vehicle movements. However, their incorporation into the model was challenging due to the unavailability or lack of detailed data. Future research should focus on generating and collecting such data to better understand their role in the risk of infection.”

For a better understanding of this phenomenon, and also to ensure a complete and objective overview, I recommend that you cite a scientific article:

Iancu, I. Tirziu, E. Pascu, C., Costinar, L., Degi, J., Badea, C., Gligor, A., Bucur, I., Popa, SA., Herman, V. EVOLUTION OF HPAI AVIAN INFLUENZA VIRUS STRAINS IN EUROPE BETWEEN 2005 AND 2023, Rev Rom Med Vet (2024) 34 | 1: 138-144, WOS:001253398200004.
<https://agmv.ro/wp-content/uploads/2024/03/138_144_Iancu.pdf>

Nicoleta Foltoș (Negrilă), G. Brănescu, V.B. Vuță, S. Bărăităreanu, Florica Bărbuceanu The circulation of highly pathogenic avian influenza viruses (HPAIV) among non-human mammals worldwide during the period 01.01.2019 – 30.04.2024 Rev Rom Med Vet (2024) 34 | 2: 27-35.
<https://agmv.ro/wp-content/uploads/2024/06/27_35_Foltos_9_C_4_11zon.pdf>

The two references have been added to the manuscript.

Reviewer #2: I am happy to see the elaboration of the manuscript. But, it has some scientific and grammatical issues in the current version, and I have highlighted some issues here in the attached MS file. Therefore, the present draft needs minor revision before further process.

The manuscript has been updated according to the minor revision.

6. PLOS authors have the option to publish the peer review history of their article ([what does this mean?](https://c05y1x9s.r.us-east-2.awstrack.me/L0/https:%2F%2Fjournals.plos.org%2Fplosone%2Fs%2Feditorial-and-peer-review-process%23loc-peer-review-history/1/010f01919ab630f5-7518b5e4-070a-4566-a57b-e47ac9bbefc6-000000/qnBMlq5IbnPXrM9I13joA3XZPig=174)). If published, this will include your full peer review and any attached files.


If you choose “no”, your identity will remain anonymous but your review may still be made public.


**Do you want your identity to be public for this peer review?** For information about this choice, including consent withdrawal, please see our [Privacy Policy](https://c05y1x9s.r.us-east-2.awstrack.me/L0/https:%2F%2Fwww.plos.org%2Fprivacy-policy/1/010f01919ab630f5-7518b5e4-070a-4566-a57b-e47ac9bbefc6-000000/XLkTJXarXtV7HHa5MLlp5hykoM4=174).

Reviewer #1: No

Reviewer #2: **Yes:** Muhammad Rizwan
